# The Role of Whole-Body Magnetic Resonance Imaging (WB-MRI) in Patients with Paroxysmal Nocturnal Hemoglobinuria (PNH)

**DOI:** 10.1038/s41598-018-31547-7

**Published:** 2018-09-07

**Authors:** Ferras Alashkar, Haemi Phaedra Schemuth, Felix Nensa, Juliane Göbel, Colin Vance, Michael Forsting, Ulrich Dührsen, Thomas Wilfried Schlosser, Alexander Röth

**Affiliations:** 1 Department of Hematology, West German Cancer Center, University Hospital Essen, University of Duisburg-Essen, Essen, Germany; 2 Department of Diagnostic and Interventional Radiology and Neuroradiology, University Hospital Essen, University of Duisburg-Essen, Essen, Germany; 3 Rheinisch-Westfälisches Institut für Wirtschaftsforschung, Essen, Germany

## Abstract

In PNH thromboembolic events (TEs) represent the leading cause of morbidity and mortality. Between Dec 2013 and Jan 2016 37 PNH patients (pts) (23 PNH, 14 AA/PNH; 51% (19/37) females; median age 44 years, median D-dimer levels 0.22 mg/l) were examined with a whole-body magnetic resonance imaging (WB-MRI) scan at 1.5 T to detect TEs. Pts were treated according to German PNH guidelines, including eculizumab therapy. 64% (24/37) of the pts had no documented TEs prior to observation. Two pts had suspected TEs in their clinical history. 29% of the pts (11/37) had a known history of venous thromboses (deep venous thrombosis (DVT) (5/11), portal venous thrombosis (PVT) (4/11), vena caval thrombosis (VCT) (2/11). A myocardial infarction was reported in one pt, and two had a cerebral venous sinus thrombosis (CVST) or a thalamic infarction. Six pts (16%) had at least two prior TEs. In pts with prior TEs no progression of the existing TEs was observed. In pts on eculizumab and prior TEs as well as treatment-naïve pts silent bone and renal infarctions were detected. Furthermore, a clinically non-critical arterial occlusion was identified. WB-MRI scans present a novel, non-invasive method to assess the complete vascular status of PNH pts and allow the detection of previously undiagnosed vascular complications, affecting treatment indications and regimens.

## Introduction

Paroxysmal noctural hemoglobinuria (PNH) is a rare, genetically acquired, non-malignant clonal disorder of the pluripotent hematopoietic stem cells characterized by the classic triad of (a) chronic, uncontrolled complement-mediated intravascular hemolysis resulting in (b) thrombophilia, and cytopenia of various degrees due to (c) bone marrow failure^1,2^. PHN results as a consequence of somatic mutations of the phosphatidylinositol glycan A (PIG-A) gene in either one or several pluripotent hematopoietic stem cells of the bone marrow. However, the PIG-A gene is essential for the biosynthesis of glycosylphosphatidylinositol anchor proteins (GPI-APs) on cell membranes. The defective GPI-anchored protein synthesis results in a reduced expression or even absence of GPI-anchored proteins such as CD55 (DAF - decay accelerating factor) and CD59 (MIRL - membrane inhibitor of reactive hemolysis), which play an essential role in complement regulation, especially affecting the alternative complement pathway, explaining the increased sensitivity of PNH cells to complement-mediated intravascular hemolysis, vascular inflammation, and systemic hemoglobin release. All of these factors account in part for the increased thrombophilic tendency in PNH patients, in addition to platelet activation, impaired nitric oxide (NO) bioavailability, impairment of the fibrinolytic system, etc.^3–5^.

These mechanisms in concert demonstrate a close correlation between the complement system and the coagulation cascade, making PNH the most known viciously acquired thrombophilic state in internal medicine^6^. Despite the fact that the PNH clone size correlates with the possibility to acquire a thrombosis, thromboses are also found in patients with small PNH clones of less than 10%^7–9^. Anticoagulation alone may not be as effective in preventing the occurrence of thromboembolic events (TEs) in PNH patients, as shown by several case reports, emphasizing the unpredictability of TEs in the setting of PNH even on chronic eculizumab treatment^10,11^. Hence, prophylactic anticoagulation still remains a controversial subject in these patients, especially in non-eculizumab-treated patients. By now, the monoclonal antibody eculizumab, which is indicated in symptomatic PNH patients, has proven to be the only effective therapeutic manner in reducing the risk for thromboses in PNH dramatically, improving survival rates of patients^2^.

In summary, TEs represent by far the leading cause of morbidity and mortality in PNH patients, accounting for approximately 22–67% of PNH-related deaths. The incidence to acquire at least one TE during the course of the disease in PNH varies from 29% to 44%^4,11–13^. Given that the rates of silent thromboses in PNH are likely underestimated^14^, the importance of a prompt and accurate diagnosis of thrombotic events by high sensitive imagining is crucial and highly implicated in PNH, influencing the clinical outcome, mortality rates, and the need for treatment. However, a whole-body magnetic resonance imaging (WB-MRI) as a screening method so far has not been implemented for patients with PNH. Usually, a diagnostic examination is performed if the patient shows clinical symptoms of a TE. The diagnostic examination is then dependent on the clinical symptoms of the patient, e.g. a patient presenting with unexplained abdominal pain and known PNH would be examined with an abdominal CT or MRI scan only. Silent TEs in other areas, however, would not be detected by the abdominal examination even though silent TEs could also result in a change of the therapeutic regimen.

Combined arterial and venous whole-body MR-angiography has been proven feasible at 1.5 T in patients with thromboembolic diseases to detect arterial and venous TEs^15^. Furthermore, a WB-MRI not only detects TEs with a combined arterial and venous whole-body MR-angiography, but also detects infarctions as a result of a prior TE.

The aim of this study was to examine the feasibility of WB-MRI to detect and monitor clinically apparent as well as silent TEs in PNH patients.

## Patients and Methods

Retrospective analysis and use of data was approved by the Ethical Committee of the Faculty of Medicine at the University Hospital of Duisburg-Essen. Informed consent for WB-MRI, including the administration of a contrast agent, was obtained from all patients. The study has been conducted in accordance with the Declaration of Helsinki and with the EU ICH GCP Guidelines. All patients had a clinically indicated WB-MRI. Exclusion criteria were any contraindications for a WB-MRI or the administration of a contrast agent.

### Patient characteristics

Between Dec. 2013 and Jan. 2016 clinically indicated WB-MRI scans were performed in 37 patients (51% (19/37) females; median age 44 years (range 24–73 years) with either PNH (n = 23) or AA/PNH-syndrome and a detectable PNH clone (n = 14) of the currently 207 patients enrolled in the prospective, observational, non-interventional PNH-Registry of the University Hospital of Duisburg-Essen.

In 67% (25/37) of the patients no TEs were documented prior to the MRI scan. Two patients had a suspected TE due to their clinical history, including pulmonary embolism (PE) (1/37) or deep venous thrombosis (DVT) (1/37). Among the remaining patients (29% (11/37)), predominantly venous thromboses (e.g. deep venous thromboses (DVT) (5/11), portal venous thromboses (PVT) (4/11), or vena caval thromboses (VCT) (2/11)) were documented prior to the examination time. Other thrombotic events included a cerebral sinus thrombosis (CSVT) (1/37) or a thalamic infarction (1/37). One patient had a history of myocardial infarction. Six patients, reflecting 16% of the cohort population, had at least two documented TEs in their medical history.

At the time of investigation serum lactate dehydrogenase (LDH) levels (median (overall) serum LDH levels 272 U/l, range 169–1530 U/l; normal range 120–247 U/l), D-dimer levels (median (overall) serum D-dimer levels 0.22 mg/dl, range <0.19–5,08 mg/dl; normal range <0.55 mg/dl), and clone sizes of GPI-deficient granulocytes measured by flow cytometric assay via fluorescein-labeled proaerolysin (FLAER) (GPI-deficient granulocytes (FLAER) 84.5% (median), range 5–100%) were obtained for each patient, respectively.

All patients were treated according to current German PNH guidelines, including the use of the terminal complement inhibitor eculizumab (70% (26/37)), with eculizumab being initiated in 23 patients prior to WB-MRI scans. In the remaining three patients, eculizumab was initiated later on for reasons other than TEs.

### Whole-Body Magnetic Resonance Imaging (WB-MRI)

All WB-MRI scans were performed at a 1.5 Tesla (T) MRI scanner (Siemens MAGNETOM® Avanto, Siemens Healthcare Solutions, Erlangen, Germany).

The standardized protocol included a cranial T2-SWI (susceptibility weighted imaging), a cranial 3D-TOF-MRA (time of flight MR-angiography), a cranial T2-SPACE (sampling perfection with application optimized contrasts using different flip angle evolution), a whole-body arterial and venous contrast-enhanced MRA and a contrast enhanced T1-weighted whole-body VIBE (volume interpolated breathhold examination).

T2-susceptibility weighted images (SWI) of the neurocranium were acquired (repetition time (TR) 48 ms; echo time (TE) 40 ms; matrix 156 × 320 mm; FOV 230 mm; flip angle 15°; acquired voxel size 1.2 × 0.72 × 2.0 mm, reconstructed voxel size 1.2 × 0.7 × 2.0 mm; slice thickness 2 mm; acquisition time 3:33 min) in transversal slices. 3D-time of flight (TOF)-angiography of the neurocranium were also acquired in a transverse plane (repetition time (TR) 26 ms; echo time (TE) 7.0 ms; matrix 211 × 256 mm, acquired voxel size 0.75 × 0.7 × 0.7 mm; reconstructed voxel size 0.7 × 0.7 × 0.7 mm; FOV 180 mm; flip angle 25°; slice thickness 0.7 mm; acquisition time 4:05 min). A cranial 3D-T2 weighted SPACE was acquired in a sagittal plane (repetition time (TR) 6000 ms; echo time (TE) 333 ms; matrix 218 × 256 mm; FOV 260 mm; TI 2200 ms; acquired voxel size 1.04 × 1.02 × 1.0 mm, reconstructed voxel size 1.0 × 1.0 × 1.0 mm; slice thickness 1.0 mm; acquisition time 7:02 min).

The whole-body contrast-enhanced arterial MR-angiography (MRA) is based on the acquisition of four overlapping 3D datasets acquired in coronal planes with a 3D fast low angle shot (FLASH) gradient recalled echo sequence with steps for the

- Head, neck, and upper chest (repetition time (TR) 2.8 ms; echo time (TE) 0.94 ms; matrix 173 × 384 mm; FOV 500 mm; flip angle 25°; acquired voxel size 2.17 × 1.3 × 1.2 mm, reconstructed voxel size 2.2 × 1.3 × 1.2 mm; slice thickness 1.2 mm; acquisition time 0:11 min).
- Lower chest and abdomen (repetition time (TR) 2.74 ms; echo time (TE) 0.9 ms; matrix 151 × 384 mm; FOV 500 mm; flip angle 25°; acquired voxel size 2.17 × 1.3 × 1.4 m, reconstructed voxel size 2.2 × 1.3 × 1.4 mm; slice thickness 1.4 mm; acquisition time 0:11 min).
- Pelvis and upper leg (repetition time (TR) 2.74 ms; echo time (TE) 0.9 ms; matrix 154 × 384 mm; FOV 500 mm; flip angle 25°; acquired voxel size 2.17 × 1.3 × 1.4 mm, reconstructed voxel size 2.2 × 1.3 × 1.4 mm; slice thickness 1.4 mm; acquisition time 0:11 min).
- Lower legs and feet (repetition time (TR) 2.88 ms; echo time (TE) 0.98 ms; matrix 211 × 512 mm; FOV 500 mm; flip angle 25°; acquired voxel size 1.63 × 0.98 × 1.20 mm, reconstructed voxel size 1.6 × 1.0 × 1.2 mm; slice thickness 1.2 mm; acquisition time 0:15 min).

The contrast agent administered was Gadovist (Gadobutrol 1.0 mmol/ml, Bayer) with a dose of 0.2 ml/kg bodyweight and an injection rate of 1.2 ml/s for the first half of the contrast agent and an injection rate of 0.7 ml/s for the second half of the contrast agent followed by a saline flush with an injection rate of 0.7 ml/s for 20 ml of 0.9% saline solution.

The whole body MR-venography was performed immediately after the arterial MRA with a fat suppressed 3D T1-weighted gradient echo volume interpolated breath hold-examination (VIBE) sequence acquired in axial planes with 8 to 10 steps depending on the patients height (repetition time (TR) 3.66 ms; echo time (TE) 1.4 ms; matrix 166 × 512 mm; FOV 480 mm; flip angle 12°; acquired voxel size 2.08 × 0.94 × 3.50 mm, reconstructed voxel size 2.1 × 0.9 × 3.5 mm; slice thickness 3.5 mm; acquisition time per step 0:20 min). The venography was acquired from the base of the skull to the veins of the feet.

The total time of the protocol was 20:10 minutes.

A DWI/ADC or FLAIR sequence of the brain was not acquired, since none of the patients examined presented with acute or subacute neurological symptoms. Instead of the FLAIR sequence a 3D SPACE sequence was used to assess white matter lesions.

All acquired MR images were then assessed by two radiologists in consensus for the clinical report and again assessed retrospectively by two radiologists (4 years in cardiovascular training and 14 years in cardiovascular training) in consensus for the presence or absence of TEs as well as infarctions as a result of a TE.

All acquired cranial MR images were also assessed by two blinded radiologists (4 years in neuroradiological training and 5 years in neuroradiological training) retrospectively for cerebral changes associated with TEs.

As suggested by Barcellini *et al*.^16^, the agerelated white matter changes (ARWMC) scale as classified by Wahlund *et al*.^17^, was used to assess and quantify chronic ischemic small vessel disease defined as white matter abnormalities with T2 hyperintense signal with a distinction between deep white matter (WM) and basal ganglia (deep grey matter). The presence or absence of diffuse T2 signal hyperintensities of the periventricular WM which were consistent with chronic vascular degeneration or leukoaraiosis was also assessed. Focal alterations consistent with old ischemic strokes were also assessed and define as malacic fluid-filled cavities presenting with a hyperintense signal on T2-weighted images. The absence or presence of active or previous bleedings or microhemorrages as well as atrophy was also evaluated.

The score for WM lesions was calculated as suggested for each side separately for the frontal, parietal-occipital, and temporal lobe, and also separately for the infratentorial compartment. WM lesions as well as basal ganglia lesions were scored as following: 0 = no lesions, 1 = focal lesion, 2 = initial confluence of lesions, 3 = broad involvement of the entire region.

For leukoaraiosis a more general score was used with 0 = absence of lesions, 1 = mild to moderate, 2 = severe.

The circle of Willis and its branches was also evaluated for abnormalities, e.g. stenosis, aneurysms, and Moya-Moya-like alterations, as well as the cerebral venous sinus for the presence or absence of thrombosis or abnormalities e.g. aplasia.

## Results

**Figure 1:**
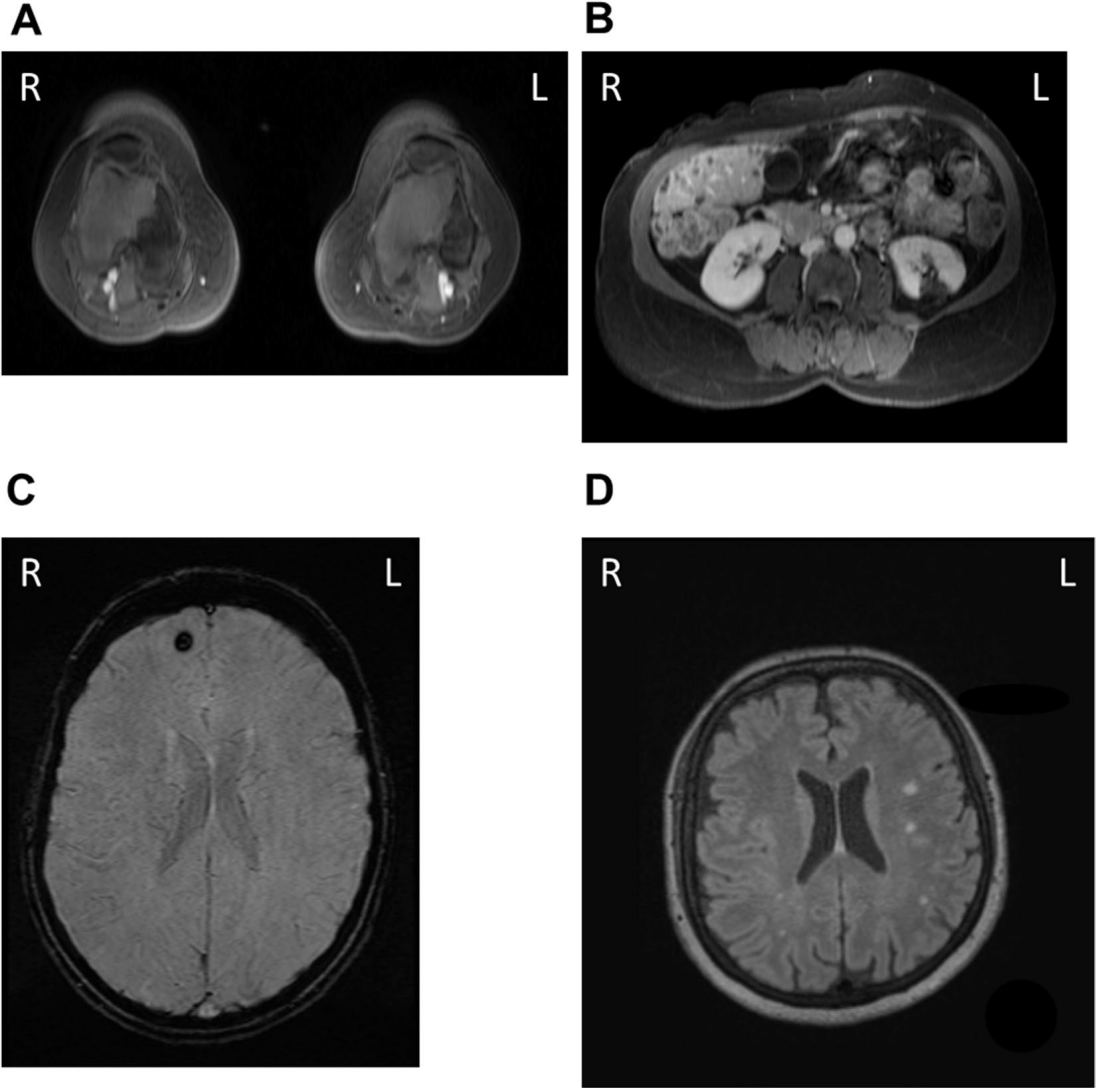
(**A**) T1-weighted, contrast-enhanced volume interpolated breathhold examination (VIBE-) images of non-symptomatic bilateral bone infarctions of the vascular bed of the lower extremities (femoral and proximal tibial bone) in a 37-year-old female PNH patient with a medical history of portal- and liver venous thrombosis (patient 26). (**B**) T1-weighted, contrast-enhanced VIBE-images of a left renal infarction in a 56-year-old female PNH patient with no medical history of thromboembolic events (patient 8). (**C**) Microbleeds in a 32-year-old female PNH/AA patient with prior history of superficial lower leg venous thrombosis (patient 18). (**D**) White Matter Changes (ARWMC scale, see Materials and Methods), leukoaraiosis, and cerebral atrophy in a 60-year-old female PNH patient (patient 29); Abbreviations: L: left; R: right.

In PNH patients with a medical (10/37) or even possible medical history (2/37) of TEs, we could not confirm a progression of the existing thrombotic events at the time of MRI examination. In 11 out of these 12 patients, eculizumab treatment was initiated prior to the WB-MRI examination (median eculizumab treatment duration 15.1 months; range 2.9–68.5 months) either due to thrombotic events (3/11) and/or other PNH-associated complications, including a significant transfusion requirement for red blood cells, recurrent abdominal pain crisis, or recurrent and severe PNH-associated hemolytic crisis. In patients on chronic eculizumab therapy and documented prior thromboses or TEs, in addition to patients with suspicion of thrombotic events in their medical history, the reported thrombotic events could be confirmed by WB-MRI in four patients (patient 2, 15, 16, and 22). Beyond this, unknown TEs prior to the examination date, including non-symptomatic bilateral bone infarctions of the vascular bed of the lower extremities (femoral and proximal tibial bone) could be detected in one patient (patient 26 - Fig. 1A) by the WB-MRI in addition to prior undiscovered bilateral renal infarctions in another patient (patient 15) with a history of multiple thromboembolic complications in the past. Moreover, in one female patient (patient 33), in whom eculizumab treatment was initiated due to a severe transfusion requirement for red blood cells, an asymptomatic bone infarction of the right proximal femur was additionally diagnosed by the MRI examination. In one male patient, in whom eculizumab was initiated due to recurrent abdominal pain crises, a previously not documented, clinical non-critical occlusion of the right anterior tibial artery was identified.

In comparison to PNH patients, in whom eculizumab treatment was not indicated by the time of the MRI examination according to current German PNH guidelines, a previously undiagnosed left renal infarction (patient 8 - Fig. 1B) as well as non-symptomatic bilateral bone infarctions of the femoral and proximal tibial bones (patient 25) could be diagnosed by MRI.

Only one patient was known to have a cerebral infarction in the history. In 22 patients white matter abnormalities relative to chronic ischemic small vessel disease could be detected, with 14 patients presenting with periventricular and deep white matter lesions, three patients with only periventricular white matter lesions, and five patients presenting with only deep with matter lesions. No intracranial arterial abnormalities were detected and no venous abnormalities distinguishable from anatomical variants were present, whereas, in five patients microbleeds or cerebral hemorrhages were detected (e.g. patient 18 or 29 - Fig. 1C,D).

In PNH patients, in whom previously unveiled thrombotic complications were identified, PNH clone size of GPI-deficient granulocytes (FLAER) was larger than 50% at the date of the MRI examination. Taking the formerly not diagnosed thrombotic findings into account, the overall number needed to treat in the setting of PNH by WB-MRI scans was 8.0 (95% CI, 4.1–27.8). Independently of the observed findings, there was no evidence of pulmonary embolism in the studied PNH patients at observation time. The complete patient characteristics at the time of examination, including the observed findings, are summarized in Table 1.

**Table 1:** Clinical Characteristics of 37 Patients with PNH or PNH/AA-Syndrome undergoing Whole-Body Magnetic Resonance Imaging (WB-MRI) Scans between Dec 2013 - Jan 2016 (University Hospital of Essen).

Patient	Diagnosis		Gender	Age	GPI-deficient gran.	LDH	D-dimer	Eculizumab initiation		Medical history of TEs	WB-MRI findings	White matter changes (ARWMC scale)						
				(yrs.)	(FLAER) (%)	(U/l)	(mg/l)					Leukoa-raiosis	Periventricular WM	Deep WM	Venous system abnorm-alities	Arterial system abnor-malities	Microbleeds/bleedings	Atrophy
				(Examination date)				Prior to MRI scans	Post MRI scans									
1	PNH	11/97	male	57	73	277	0.26	10/09		05/00 left DVT	—	mild to moderate	4	4	no	no	no	yes
2	AA/PNH	01/14	female	73	5	267	5.08	—	—	possible clinical history of caval and right femoral thrombosis (DVT)	confirmation of inf. caval (MRI) and right femoral thrombosis (USS)	mild to moderate	0	3	no	no	yes	yes
3	AA/PNH	84	male	44	99	253	0.21	10/11		—	—	absent	0	0	no	no	no	no
4	PNH	08/13	male	24	86	265	0.19	09/13		—	—	absent	0	0	no	no	no	no
5	PNH	03/14	female	62	89.3	1530	0.47	—	04/14	—	—	mild to moderate	2	1	no	no	no	yes
6	PNH	02/13	male	32	92	243	<0.19	07/13		—	clinical non-critical occlusion of the right distal anterior tibial artery	absent	0	0	no	no	no	no
7	AA/PNH	03/13	female	64	6.9	364	0.49	—	—	—	—	mild to moderate	3	1	no	no	yes	yes
8	PNH	01/14	female	56	63	393	<0.19	—	07/15	—	left renal infarction	absent	0	0	no	no	no	yes
9	PNH	02/02	female	55	98.2	284	0.19	11/10		10/10 bilateral DVT	—	mild to moderate	1	3	no	no	no	no
10	PNH	06/96	female	66	65	1429	0.63	—	11/14	—	—	mild to moderate	4	3	no	no	no	yes
11	PNH	06/14	female	58	98	211	0.39	10/12		—	—	mild to moderate	0	1	no	no	no	yes
12	PNH	10/10	male	30	64	1051	<0.19	—	—	—	—	absent	0	0	no	no	yes	no
13	AA/PNH	01	female	29	100	196	0.26	04/13		05 cerebral venous sinus thrombosis, 05/11 portal venous thrombosis	—	absent	0	0	no	no	no	no
14	PNH	09/09	male	24	83	1025	0.23	—	—	—	—	absent	0	0	no	no	no	no
15	PNH	88	female	51	96	284	<0.19	11/08		06/05 right subclavian venous and vena cava superior thrombosis, 07 and 08 port catheter thrombosis	bilateral renal infarction, confirmation of right subclavian, brachiocephalic & sup. vena cava thrombosis, thrombosis of the left v. iliaca externa	mild to moderate	1	2	no	no	yes	yes
16	PNH	01/14	male	52	68	272	0.59	01/14		01/14 thalamic infarction, portal venous and lienal venous thombosis, right DVT	confirmation of lienal venous thrombosis with complete recanalization of the portal vein	mild to moderate	5	6	no	no	no	yes
17	AA/PNH	04/09	female	37	62.5	562	<0.19	—	—	—	—	mild to moderate	4	0	no	no	no	no
18	PNH	11/11	female	32	99	253	0.21	02/12		superficial lower leg venous thrombosis	—	absent	0	0	no	no	yes	no
19	AA/PNH	01/11	male	27	91	265	<0.19	04/12		—	—	absent	0	0	no	no	no	no
20	AA/PNH	06/97	male	49	30	524	0.3	—	—	—	—	mild to moderate	4	0	no	no	no	no
21	PNH	05/03	male	36	88	230	<0.19	12/04		—	—	absent	0	0	no	no	no	no
22	PNH	07/13	female	31	80	226	<0.19	08/13		03/13 portal venous thrombosis,	cavernous transformation of the portal vein	absent	0	0	no	no	no	no
										04/13 late abort	(CTPV)							
23	PNH	09	male	38	100	330	0.19	08/14		02/12 myocardial infarction	—	mild to moderate	1	1	no	no	no	no
24	PNH	09/06	male	44	46	240	0.22	11/10		—	—	mild to moderate	1	2	no	no	no	yes
25	AA/PNH	11/12	male	72	57	632	0.6	—	—	—	bilateral bone infarcts (femur/prox. tibia)	mild to moderate	3	5	no	no	no	yes
26	PNH	10/99	female	37	100	184	0.64	08/09		02/08 portal- and liver venous thrombosis	bilateral bone infarcts (femur/prox. tibia)	mild to moderate	0	4	no	no	no	no
27	AA/PNH	09/10	female	47	42	482	0.6	—	—	—	—	mild to moderate	2	4	no	no	no	no
28	PNH	06/09	male	29	99	169	0.19	04/11		—	—	absent	0	0	no	no	no	no
29	AA/PNH	02/07	female	33	11.4	257	0.36	05/14		05/14 left DVT	—	mild to moderate	1	0	no	no	no	yes
30	AA/PNH	10/13	female	40	33	538	0.21	—	—	—	—	mild to moderate	6	4	no	no	no	yes
31	PNH	03/10	male	44	100	285	0.33	03/10		—	—	mild to moderate	1	2	no	no	no	yes
32	AA/PNH	09/01	female	56	100	247	0.25	04/08		—	—	mild to moderate	0	4	no	no	no	yes
33	PNH	03/12	female	60	100	216	0.21	03/12		—	bone infarct (right prox. femur)	mild to moderate	2	5	no	no	no	yes
34	PNH	02/13	male	42	55.2	264	0.21	03/13		—	—	mild to moderate	1	0	no	no	no	yes
35	AA/PNH	01/15	male	45	2.6	258	<0.19	—	—	—	—	mild to moderate	0	4	no	no	no	yes
36	PNH	05/15	male	37	78	283	0.32	05/15		possible clinical history of PE	—	absent	0	0	no	no	no	yes
37	AA/PNH	02/15	female	27	76	527	0.25	—	—	—	—	absent	0	0	no	no	no	no

**Reference values:** LDH: 120–247 U/l; D-dimer: < 0.55 mg/l.

**Abbreviations:** AA: aplastic anemia; ARWMC Scale: age-related white matter changes Scale; CTPV: cavernous transformation of the portal vein; DVT: deep venous thrombosis; FLAER: fluorescein-labeled proaerolysin; GPI-deficient Gran.: glycosylphophatidyl-inositol deficient granulocytes; LDH: lactate dehydrogenase; PE: pulmonary embolism; PNH: paroxysmal nocturnal hemoglobinuria; TE: thromboembolic events; USS: ultrasound scan; WM: white matter; WB-MRI: whole-body magnetic resonance imaging; yrs: years.

## Discussion

In PNH patients, thromboembolism represents the most common cause of morbidity and mortality^18^. In these patients thromboses may occur in typical as well as atypical locations, affecting in particular the venous system, especially hepatic (Budd-Chiari syndrome) and cerebral venous vessels^1,19^. Beyond that, the occurrence of visceral and intraabdominal venous thromboses with involvement of mesenteric, portal, and splenic veins, in addition to vena cava and deep venous thromboses, are found to be more frequent in the setting of PNH in comparison to the general population^8,20^. Apart from venous thromboses, arterial thromboses are also more frequently found in these patients, leading to the occlusion of cerebral, coronary, visceral, and retinal vessels with subsequent infarctions^9,21–23^.

As depicted by multiple studies in the past and summarized by Hill *et al*. in 2013, the presence of TEs and their associated complications is of high prognostic value and affiliated with a poor survival (relative risk at 8 years, 10.2)^4^. Likewise, a survival rate of 40% at four years was computed by Socié *et al*., 1996, in the context of thrombosis at initial presentation prior to the eculizumab-era, in addition to an up to five- to 15.4-fold overall increased risk of death as shown by Nishimura *et al*. (2004) in patients with PNH in the United States and Japan^12,24^. It is not surprising, that the rate of silent thrombosis, even in patients with recent disease onset, seems to be underestimated, as proven by the work of Hill *et al*., 2012, providing evidence of previous subclinical pulmonary thromboses in addition to subclinical myocardial damage in the presence of PNH, respectively^14^. Therefore, silent thrombosis and TEs, if not detected promptly, may subsequently result in permanent organ dysfunction, significantly restricting quality of life.

The current retrospective monocentric study performed at the Department of Hematology at the University Hospital of Essen between Dec 2013 and Jan 2016 was designed to evaluate the rates of subclinical and silent venous and arterial thromboembolic complications in otherwise clinical asymptomatic PNH patients and also to follow up on known TEs by the use of WB-MRI scans. To the best of our knowledge, this is the first report in PNH patients for whom the complete vascular status at a given point in time was subject to analysis. As described in the patient characteristics, 37 patients with either classic PNH or a detectable PNH-clone were assessed with a WB-MRA. Out of these 37 patients, 62% of the patients (23/37) were on current eculizumab treatment according to current German PNH guidelines by the time of examination. Twelve out of these 23 patients (52%) had a medical history of prior thrombotic events. In three of these twelve patients (25%), thrombotic events preceded the diagnosis of PNH. In one of these three patients, a diagnosed portal venous thrombosis in addition to a late abort led to the diagnosis of PNH (patient 22). In the second patient a possible medical history of pulmonary embolism prior to the diagnosis of PNH was evident (patient 36). The third patient had a possible history of a caval and right femoral venous thrombosis prior to the diagnosis of PNH (patient 2), which was confirmed by duplex sonography and the acquired MR images. In three of the remaining 14 patients, eculizumab treatment was initiated for other reasons than thrombotic events, while in the rest of the patients, eculizumab therapy was not indicated at the time of examination as they remained asymptomatic or were under current immunosuppressive therapy due to bone marrow failure at the time of examination.

Assuming the initial hypothesis of silent thrombosis in the setting of PNH, the observed data are consistent with other studies, supporting the evidence of previously not-recognized thrombotic events^14^. Our findings demonstrate the presence of additional silent renal infarctions of unknown age in five percent of the patients (2/37). Out of these patients, one patient had a history of multiple thrombotic events and therefore was already under chronic eculizumab treatment (patient 15), whereas in the second patient, no prior thrombotic complications had been documented in the past, so far being treatment-naïve. In contrast to previous studies there was no evidence for silent major TEs (e.g. PE) at the time of analysis^14^.

In addition to previously not-diagnosed renal infarctions, bone infarctions of unknown age of the lower extremities, affecting both the femoral and proximal tibial bones, could be detected by WB-MRI scans in three patients (8%). At this point of time, bone infarctions represent a novel embolic complication in the context of PNH. As the overall prevalence of bone infarctions is still unknown, several risk factors have been identified, with glucocorticoid therapy, chronic alcohol abuse, and dyslipidemia representing the most common causes predisposing to their development. Other risk factors include an antiphospholipid syndrome or hemoglobinopathies such as sickle-cell disease (SCD) and thalassemia^25^. However, bone infarctions are reported as rare or very rare. As the empirical treatment of PNH involves the use of steroids, despite the fact that studies concerning the role of glucocorticoid therapy in PNH are inconsistent and randomized trials are lacking, two (patient 25 and 26) out of these three patients in whom bone infarcts were found to be present, were subject to intermittent steroid treatment in their past, which might have contributed to the development of our current findings of osseous infarctions. Similar to the findings of Lafforgue *et al*., 2016, the distal femoral and proximal tibial bones represent the most common sites of involvement.

With the help of this high sensitive and fast whole body imaging technique, an otherwise not recognized non-critical occlusion of the right anterior tibial artery could be identified in a male patient, in whom eculizumab treatment had been initiated due to hemolytic and recurrent abdominal pain crisis (patient 6).

However, to detect myocardial scarring after myocardial infarction would have required a cardiac MRI including late gadolinium enhanced (LGE) sequences. As adding a cardiac MRI (cMRI) would have lengthened the examination time for the patient, the protocol did not include a cMRI. Therefore, the number of silent myocardical infarctions could not be assessed and may be underestimated in PNH patients.

Also the number of patients with pulmonary infarctions may also be underestimated due to the CT being the gold standard to diagnose changes in the pulmonary tissue. However, pulmonary embolisms would have been detected with a specificity of up to 87% by the MRI as shown by Hu *et al*. in 2016^26^.

Recently Barcellini *et al*., 2017, investigated for the first time brain ischemic involvement in neurologically asymptomatic PNH patients^16^. In five of our patients microbleeds or signs for cerebral hemorrhage could be detected. However, the clinical value remains unclear, as none of these patients had a known history of traumatic brain injury.

A limitation of this study is, that none of the patients had been evaluated neurologically or psychologically by a certified neurologist prior to examination. However, only one of the patients included in this study had prior reported neurological symptoms. Like Barcellini *et al*., 2017, reported white matter lesions could be detected in our patients. White matter abnormalities were found in 59.5% (22/37) of our patients, whereas Barcellini *et al*. only reported 32%. The value of this finding remains unclear. Atrophy was also detected in 20 (54%) of our patients. However, as stated before, further psychological and neurological testing should be warranted to detect if this can be correlated to neurological and psychological functions. Also acute cerebral ischemic lesions might not be found, because a DWI was not performed. However none of the patients examined reported any acute neurological symptoms.

This study further provides evidence of silent and underdiagnosed thromboembolic complications in PNH patients. This is the first description of bone infarctions as possible TEs in PNH. WB-MRI scans thus seem to be a novel and feasible method for the assessment of the whole vascular status of a PNH patient and allow the detection of previously unidentified vascular complications and follow up on known thromboembolic complications. This might directly affect the treatment regimen as eculizumab is indicated in PNH patients with TEs. The question arises as to whether WB-MRA should be considered the gold standard at the time of PNH-diagnosis to determine eculizumab-treatment. Further studies should examine if silent TEs also have an impact on the mortality and morbidity of PNH-patients and therefore should be also considered as an indication to start treatment with eculizumab. In patients with existing TEs, WB-MRI scans might be employed to document their impact prior to treatment, especially in patients with major TEs or in patients with a high disease activity and suspicion for TEs (e.g. recurrent abdominal pain or persisting dyspnea).

## Data Availability

All data generated or analysed during this study are included in this published article (and its Supplementary Information files).
